# Circulating Biomarkers and Targeted Therapy in Pleural Mesothelioma

**DOI:** 10.3390/cancers17233863

**Published:** 2025-12-01

**Authors:** Christopher R. Grant, Lyudmila Bazhenova, Karen M. Yun

**Affiliations:** Division of Hematology-Oncology, Moores Cancer Center at UC San Diego Health, San Diego, CA 92093, USA

**Keywords:** pleural mesothelioma, targeted therapy, biomarker

## Abstract

Pleural mesothelioma (PM) is a rare and aggressive cancer. Standard of care for unresectable disease consists of chemotherapy, vascular endothelial growth factor inhibition, immunotherapy, or combinations thereof. Despite recent therapeutic advances, validated diagnostic and prognostic biomarkers are lacking, and no targeted therapies have been approved by the U.S. Food and Drug Administration (FDA) to date. Given the growing body of research investigating molecular alterations in PM, this review summarizes emerging diagnostic and prognostic biomarkers, discusses potentially targetable molecular alterations, and examines ongoing trials directed at targeting specific molecular mechanisms in this disease.

## Simple Summary

Pleural mesothelioma is a difficult cancer to diagnose. Several biomarkers have been studied to help diagnose patients and monitor treatment response. One biomarker is approved but not commonly used in clinical practice. Pleural mesothelioma is also an aggressive cancer that is not surgically resectable for most patients who are diagnosed with it. Traditionally, chemotherapy, immunotherapy, or a combination of both is the standard of care for patients with advanced pleural mesothelioma. Many clinical trials have been performed to target mutations in mesothelioma, but none have been approved for treatment. In this article, we review potential circulating biomarkers and targeted therapy clinical trials that have occurred in pleural mesothelioma. We also discuss ongoing clinical trials and potential future targeted therapy treatment options for pleural mesothelioma.

## 1. Introduction

Pleural mesothelioma (PM) is an aggressive cancer originating from the mesothelial cells of the pleural viscera [1]. PM has an incidence of 2803 new cases per year in the United States [2]. PM is diagnosed by tissue biopsy and classified into three major histologic subtypes: epithelioid, sarcomatoid, and biphasic. Traditionally, patients with PM can be treated with a multimodal approach that includes systemic therapy and, in some cases, surgical resection or radiation therapy. The current role of surgery in PM is controversial. Surgical resection is typically reserved for those with epithelioid histology, node-negative disease, and minimal comorbidities [3]. While there is a goal of complete cytoreduction and negative margins similar to other cancers like colon or lung cancer, mesothelioma’s diffuse growth pattern along the pleura makes curative surgical resection difficult. Thus, cytoreductive surgery is aimed at removing macroscopic tumors to improve disease burden. Chemotherapy is usually administered in the induction or adjuvant setting as part of a multimodal therapeutic strategy. Unfortunately, the majority of patients present with unresectable PM, which carries a poor prognosis with a 5-year survival rate of approximately 11% [4].

For patients with unresectable disease, the mainstay therapy over the past two decades has been platinum plus pemetrexed-based chemotherapy. The Mesothelioma Avastin Pemetrexed Study (MAPS) demonstrated improved overall survival (OS) with the addition of bevacizumab to chemotherapy compared to chemotherapy alone, leading to the 2019 FDA approval of bevacizumab in combination with platinum and pemetrexed for unresectable PM [5]. More recently, the advent of immune checkpoint inhibitors has transformed the treatment paradigm for unresectable PM. The combination of an anti-programmed cell death 1 (PD-1) antibody, nivolumab, and an anti-cytotoxic T-lymphocyte-associated antigen 4 (CTLA-4) antibody, ipilimumab, in CheckMate-743 (also known as IND.227) demonstrated superior OS compared to carboplatin and pemetrexed, resulting in FDA approval of dual checkpoint inhibitor therapy in 2020 [6]. Notably, a greater survival benefit was observed with immunotherapy in patients with non-epithelioid histology, a subtype historically associated with less favorable outcomes. Most recently, the Keynote-483 trial evaluated first line platinum-based chemotherapy in combination with pembrolizumab in individuals with PM and demonstrated an improved median OS compared to platinum-based chemotherapy alone, leading to the FDA approval of pembrolizumab in combination with chemotherapy in 2024 [7]. Despite these advances in systemic therapy, outcomes for PM remain poor, underscoring the urgent need for better diagnostic, prognostic, and predictive tools.

As a result, recent research has increasingly focused on identifying novel serum and pleural biomarkers in PM. Circulating biomarkers serve four foundational purposes: screening individuals at risk, improving the diagnostic accuracy, predicting prognosis, and assessing treatment response. This review will summarize emerging biomarkers in PM and provide an update on ongoing clinical trials evaluating targeted therapies in this disease.

## 2. Methods

This review was conducted using multiple databases, including PubMed, ClinicalTrials.gov, and Google Scholar. Studies that were included consisted of prospective and retrospective analyses evaluating potential diagnostic, prognostic, and predictive circulating biomarkers for pleural mesothelioma, as well as prospective studies investigating potential targeted therapies. The following search terms were used in the literature search in PubMed and Google Scholar: “mesothelioma,” “pleural mesothelioma,” “biomarker,” and “targeted therapy.” When evaluating ongoing clinical trials through ClinicalTrials.gov, the following search terms were used: “mesothelioma” and “pleural mesothelioma.” Data extracted included the name of the author, publication year, study characteristics, outcomes including sensitivity and specificity for circulating biomarkers as well as overall response rate (ORR), progression free survival (PFS), disease free survival (DFS), and OS for all studies when available. Once a circulating biomarker or systemic therapy was discovered during the literature search, an abbreviated literature review was performed to provide background.

## 3. Circulating Diagnostic Biomarkers

**Figure 1:**
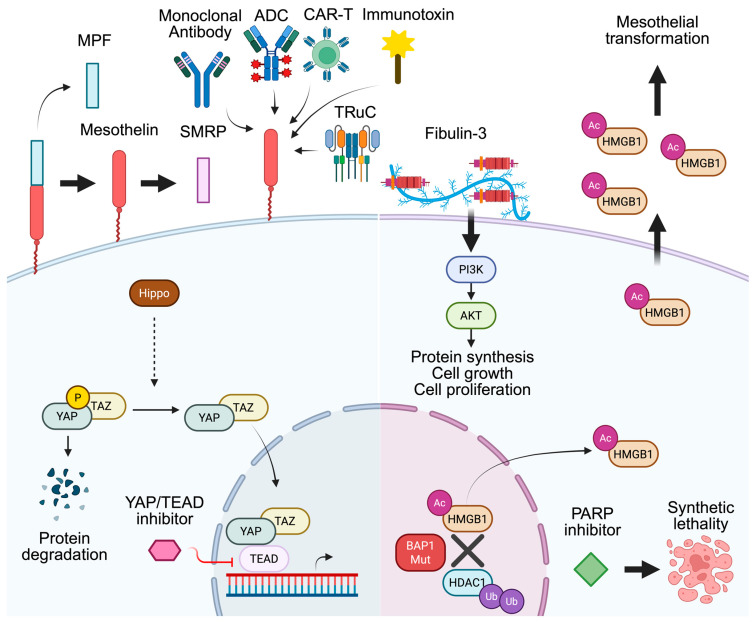
Highlighted schematic diagram of biologic mechanisms for circulating biomarkers and molecular targets. Figure created with BioRender Grant. (2025) https://BioRender.com/8gvjmoj [18]. Abbreviations: Ac, acetylation; ADC, antibody drug conjugate; AKT, Ak strain transforming; BAP1, BRCA-1 associated protein 1; CAR, chimeric antigen receptor; HDAC, histone deacetylase; HMGB1, high-mobility group box 1; MPF, megakaryocyte potentiating factor; P, phosphorylation; PARP, poly (ADP-ribose) polymerase; PI3K, phosphatidylinositol 3-kinase; SMRP, soluble mesothelin related protein; TAZ, transcriptional co-activator with PDZ-binding motif; TEAD, TEA domain transcription factor; TRuC, T cell receptor fusion construct; Ub, ubiquination; YAP, yes-associated protein.

Non-invasive biomarkers have the potential to assist in early diagnosis, inform prognosis, and help predict response to therapy. Several such biomarkers have been investigated (Table 1) with highlighted markers depicted in a schematic diagram (Figure 1). Among them, soluble mesothelin-related protein (SMRP) is the only FDA-approved biomarker for mesothelioma. It is authorized for treatment response monitoring, but not for diagnostic use [8]. PM often presents with a patchy distribution of tumor growth, which can complicate the selection of a biopsy site [9]. Even when tissue is obtained, samples may be of low tumor cellularity or insufficient quality, limiting the accuracy of histopathologic analyses. Pathologists need adequate tissue to perform broad immunohistochemical (IHC) staining to differentiate PM from reactive mesothelium, lung adenocarcinoma, and other malignancies. Histologic subtypes can be misclassified if an adequate sample of tissue is not obtained. A potential circulating diagnostic biomarker could benefit in aiding in the diagnosis of PM as well as determining the histologic subtype.

### 3.1. Mesothelin and Soluble Mesothelin-Related Proteins

Mesothelin is the most investigated biomarker in PM. It is a cell-surface glycoprotein expressed on mesothelial cells in the pleura, peritoneum, and pericardium that functions to facilitate cellular adhesion. Overexpression of mesothelin enhances cellular adhesion, facilitating local tumor invasion and the development of mesothelioma [19]. Both pleural and serum mesothelin levels can be quantified in patients with PM. Pleural mesothelin levels are usually found in cytology-positive PM but are typically low in non-malignant conditions, such as reactive mesothelial proliferation [20]. Pleural mesothelin was first identified using the murine monoclonal antibody K1 in a small study of 23 patients with cytologically positive PM in which all 15 patients with the epithelioid subtype had expression while all 8 patients with the sarcomatoid subtype lacked expression [21]. The consistent lack of mesothelin expression in sarcomatoid PM has since been confirmed in additional studies [21,22]. Mesothelin expression in only epithelial subtypes presents an issue in its role as a diagnostic biomarker since sarcomatoid subtypes could not be reliably identified. Additionally, pleural fluid sampling can be difficult in patients with minimal pleural fluid or yield a false negative cytology. Serum mesothelin was also studied as a diagnostic biomarker for PM and was found to have a sensitivity of 23% at a predefined specificity of 99% [10]. The sensitivity modestly improved to 46% when combined with serum calretinin in a combined biomarker signature. Serum mesothelin levels assisted in differentiating 217 patients with stage I or II epithelioid and biphasic mesothelioma from 1612 high-risk controls with a sensitivity of 32%. The poor sensitivity of serum mesothelin, even in combination with serum calretinin, limits its ability to be used as a diagnostic biomarker.

SMRP are mesothelin fragments cleaved into the blood stream and thus have no direct role in the pathogenesis of PM. Variability in cut-off values have made interpretation of SMRP as a diagnostic biomarker challenging. One systematic meta-analysis evaluated the diagnostic value of serum mesothelin across 16 studies [11]. Using a threshold of 2.00 nmol/L, sensitivity and specificity were recalculated for each study. The pooled analysis demonstrated an average sensitivity of 47% (range, 19% to 68%), and an average specificity of 96% (range, 88% to 100%). The relatively low sensitivity of SMRP and biologic variability due to influencing factors limits its utility as a diagnostic serum biomarker. Consistent with this, several prospective studies have failed to demonstrate the benefit of serum mesothelin or SMRP levels as screening tools in individuals with occupational asbestos exposure [23,24].

SMRP levels have been evaluated as part of multiple biomarker panels in combination with other candidate biomarkers. One study evaluated the combined use of SMRP and serum osteopontin levels as diagnostic biomarker signature for PM, demonstrating a sensitivity of 80% and a specificity of 91.2% [25]. Mesothelin gene polymorphisms may also play a potential role in the diagnosis of PM. Specifically, the presence of the mesothelin *rs1057147* polymorphism, when combined with elevated SMRP levels, improved diagnostic specificity from 88.5% to 92.7% [12]. These two biomarker signature studies were all retrospectively designed, which does risk inherent selection bias. Further prospective analysis of the biomarker signatures will be pertinent in validating these findings.

### 3.2. Megakaryocyte Potentiating Factor

Megakaryocyte potentiating factor (MPF) is a soluble protein produced by the cleavage of the mesothelin precursor [26]. It is expressed on normal mesothelial cells as well as mesothelioma and ovarian cancer cells. MPF was initially discovered due to its ability to potentiate megakaryocyte colony formation; however, its pathophysiology in relation to PM is unknown [27]. Pleural effusion MPF levels are elevated in individuals with cytologically positive PM compared with those with other malignancies [28]. However, no significant difference has been observed between MPF levels in PM and benign effusions, limiting its use as a diagnostic biomarker. Serum MPF demonstrated modest performance at a cutoff of 20 nmol/L with a sensitivity of 52% at a predefined specificity of 95% as a diagnostic biomarker [28]. This low sensitivity is comparable to SMRP and mesothelin’s diagnostic sensitivity and limits its value as a diagnostic biomarker due to the high false-negative rate.

### 3.3. Fibulin-3

Fibulin-3 functions as a secreted extracellular matrix glycoprotein and modulates the extracellular membrane [29]. It promotes pleural mesothelioma growth through the activation of the PI3K/AKT signaling pathways to promote cellular adhesion, motility, and invasion [30]. It is widely expressed across various tissues with upregulated expression observed in cervical cancer, glioblastoma, and osteosarcoma, whereas downregulated expression is seen in prostate, lung, breast, colon, liver, and nasopharyngeal cancers. Fibulin-3 levels in pleural fluid were found to be significantly elevated in individuals with cytologically positive PM compared with those with benign pleural effusions [31]. Plasma fibulin-3 levels demonstrated a sensitivity of 96.7% and a specificity of 94.1% at a cutoff value of 46.0 ng/mL as a diagnostic biomarker for individuals with PM [32]. However, subsequent studies have not confirmed the utility of serum fibulin-3 alone as a reliable diagnostic biomarker [13,33]. The inconclusive results of fibulin-3 studies make it unreliable as a diagnostic biomarker. Combining serum fibulin-3, mesothelin, and high-mobility group box (HMGB1) into a composite biomarker improved diagnostic performance, yielding a sensitivity of 96% and specificity of 93% [13]. This cohort was small, with only 26 patients, which limits its diagnostic applicability. A subsequent meta-analysis demonstrated slightly decreased but similar findings with a sensitivity of 74% and specificity of 89% as a diagnostic biomarker signature for PM [34]. While the sensitivity of the meta-analysis was less than the initial prospective study, this discrepancy could be attributed to the heterogeneity of the included studies and selection bias. Further analysis to assess this biomarker signature in prospective trials is necessary.

### 3.4. High-Mobility Group Box 1

HMGB1 is a damage-associated molecular pattern protein that induces an immune response by signaling cellular distress, leading to cell death. Asbestos fibers cause mesothelial cell necrosis, thereby releasing HMGB1 leading to inflammation that promotes malignant transformation and mesothelioma growth [35]. BAP1 (BRCA-1 associated protein) and histone deacetylase 1 (HDAC1) have also been associated with acetylation of HMGB1 leading to secretion to promote mesothelial cell transformation [36]. Serum HMGB1 levels have demonstrated promise as a diagnostic biomarker in PM with a sensitivity of 100% and specificity of 80.28% at a cutoff of 52.16 ng/mL through a prospective trial of individuals with a long history of asbestos industry exposure in southeast China [37].

### 3.5. CD157

CD157 is a cell-adhesion glycoprotein expressed in 85% of PM. In a cohort of 295 patients with PM and non-mesothelioma tumors, CD157 pleural fluid levels demonstrated a sensitivity of 62.3% and a specificity of 73.93% with a cutoff of 23.66 ng/mL as a diagnostic biomarker for PM [38]. While this data is promising, the study was a retrospective analysis that may have inherent selection bias. The study did not distinguish if the patients evaluated had mesothelioma cytology-positive or cytology-negative effusions which could also be pertinent in understanding the role of CD157 as a biomarker. Future prospective studies will need to be performed and incorporate PM cytology into their design to better define the potential of CD157 as a diagnostic biomarker.

### 3.6. Circulating Tumor DNA

Circulating tumor DNA (ctDNA) are fragments of DNA originating from tumor cells and may serve as potential biomarkers. A study of 75 pleural fluid and matched serum samples evaluated the potential utility of DNA repeats 115 bp and 247 bp as well as the 247/115 bp ratio in the diagnosis of cytologically negative PM [14]. The pleural fluid DNA 247/115 bp ratio was elevated in individuals with PM when compared to those with benign effusions at a sensitivity of 81% and specificity of 87%. In another study, a composite biomarker signature combining thrombomodulin promoter methylation, microRNA (miRNA) 126, and SMRP was evaluated in asbestos-exposed individuals [39]. The study demonstrated a sensitivity of 60% and a specificity of 81% as a diagnostic biomarker signature. While these findings suggest that ctDNA may hold promise as a non-invasive diagnostic biomarker, further prospective studies are needed to validate these results in larger more well-defined patient populations, standardized pre-analytical and analytical methodologies, and to demonstrate reproducible findings across different disease stages and treatment settings.

### 3.7. Calretinin

Calretinin is a calcium-binding protein that functions in neuronal synaptic transmission, regulating proliferation, and stimulating apoptotic signaling [40]. It activates the focal adhesion kinase signaling pathway and promotes the epithelial to mesenchymal transition. Calretinin is expressed on the surface of mesothelial cells and can be elevated in the serum, plasma, and pleural effusions in PM [41]. In one retrospective study, serum calretinin demonstrated diagnostic utility with a sensitivity of 68.3% and a specificity of 88.6% at a cutoff value of 0.32 ng/mL [42]. Elevated serum calretinin levels were also associated with poor prognosis, correlating with a shorter median PFS and median OS [43]. These findings need to be confirmed through prospective analysis before clinical application.

### 3.8. Micro-RNA

Micro-RNA s(MiRNA) are small non-coding RNA molecules encoded by the eukaryotic genome that regulate cellular processes by repressing translation or promoting degradation of mRNA [44]. Dysregulation of miRNA can play a role in cell proliferation. One retrospective analysis found that four miRNAs (miR-19b, miR-26a, miR-26b, and miR-29a) had elevated expression levels in 85 PM patients when compared to asbestos-exposed individuals with normal tissues [45]. In particular, MiR-29a and miR-19b were upregulated in individuals with PM compared to those with non-small cell lung cancer (NSCLC). A separate retrospective study showed that elevated miR-98 expression levels have been found in individuals with PM [46]. Although these findings suggest a potential diagnostic role for specific miRNA signatures, further prospective studies are needed to confirm these results in larger, independent cohorts with standardized assay methodologies.

### 3.9. Cytokeratin 19 Fragment Antigen 21-1

Cytokeratin 19 fragment antigen 21-1 (CYFRA-21-1) is a soluble fragment of cytokeratin 19 typically present on benign epithelial tissues and absent in mesothelial tissue [47]. CYFRA 21-1 is released when cells undergo turnover through necrosis or apoptosis leading to elevated levels in PM. One study evaluated pleural fluid levels of CYFRA 21-1 and carcinoembryonic antigen (CEA) as a diagnostic signature in 106 patients, 23 of whom had cytologically positive PM [48]. The combination index had a sensitivity of 87.5% and a specificity of 3.1% using cutoff levels of 41.9 ng/mL for CYFRA 21-1 and 5.0 ng/mL for CEA. The low specificity makes this limited the diagnostic discrimination needed for a reliable biomarker. Most recently, positive *SHOX2* and *PTGER4* methylation combined with elevated serum CYFRA 21-1 levels was studied as a diagnostic biomarker signature in 48 PM patients [15]. The study found a sensitivity of 91.3% and specificity of 97.6% when differentiating PM from benign mesothelial hyperplasia at an unreported cutoff. This sensitivity and specificity is impressive and further validation in prospective trials with larger cohorts is needed to validate the diagnostic utility.

### 3.10. Activin A

Activin A is a transforming growth factor associated with inflammation-induced secretion, and suppression is found to prevent tumor angiogenesis, cancer motility, and fibrosis [49,50]. Plasma activin A levels demonstrated a sensitivity of 76% and a specificity of 73% at a cutoff level of 0.488 ng/mL when serving as a diagnostic biomarker for PM [51]. This was across epithelial, sarcomatoid, and biphasic histologic subtypes. Plasma samples were collected at time of diagnosis or prior to surgical resection; however, 45 healthy individuals served as controls, which may have presented as an inherent selection bias in the control cohort.

### 3.11. Podoplanin

Podoplanin is a type I transmembrane sialomucin-like glycoprotein that induces platelet aggregation to protect tumors from immune surveillance which causes further cellular migration and dissemination [52]. One study found podoplanin IHC expression on all 5 samples of mesothelioma tumors and negative on 118 samples of other tumors including adenocarcinomas, squamous cell carcinomas, gastrointestinal stromal tumors, and endocrine tumors [53]. Given the elevated specificity and sensitivity in vitro, podaplanin expression was further validated through a rat anti-human podoplanin Ab, coined NZ-1, in vitro and in xenograft models [54]. The study found an expression of podoplanin in 73% of PM cell lines and in 92% of PM tissues with an ability to accumulate in PM xenograft models. While this study demonstrated promising results for podaplanin expression, studies in patients are needed to demonstrate its expression in PM and its role as a biomarker or therapeutic target.

### 3.12. What Remains to Be Addressed

At this time, there are several questions that remain for diagnostic biomarkers. Circulating diagnostic biomarkers need to be defined by their clinical role such as for screening of asbestos-exposed populations or for those presenting with findings suspicious for PM, such as pleural thickening or a pleural effusion. Current existing biomarkers lack the diagnostic sensitivity and specificity necessary to detect disease in either scenario, especially for early stage disease or for non-epithelioid subtypes. Many of these studies have assessed single circulating biomarkers through large retrospective analyses. There is a paucity of data assessing biomarker signatures combining proteins, ctDNA, gene methylation, and miRNA panels. Of the few small studies of biomarker signatures that have shown promise (such as SHOX2 and PTGER4 methylation in combination with CYFRA 21-1), there is a lack of large multicenter prospective studies that are imperative to validate findings from these small retrospective analyses. Additionally, many pleural diagnostic biomarkers are studied in cytologically positive PM. Further exploration of pleural diagnostic biomarkers in cytologically negative patients would greatly improve the ability to diagnose patients and aid a large unmet clinical need.

## 4. Circulating Prognostic Biomarkers

Assessing disease progression in PM can be difficult. Response Evaluation Criteria In Solid Tumors (RECIST) was designed for easily measurable tumors with a round radiographic appearance. Standard RECIST is unreliable in PM because the disease presents as a sheet-like pleural thickening rather than discrete, measurable nodules [9]. Given the limitations of standard RECIST in assessing treatment response, the modified RECIST (mRECIST) criteria for PM was developed [55]. mRECIST for PM quantifies tumor burden by measuring pleural thickness perpendicular to the chest wall at multiple standardized levels of disease involvement. However, despite the refinements, the mRECIST is still limited by interobserver variability and inconsistencies in measurement techniques [56,57]. SMRP can assist with disease monitoring in specific clinical situations. For instance, downtrending SMRP levels can suggest treatment response. Despite this, SMRP is not routinely used in clinical practice due to its limited reliability, characterized by low sensitivity for PM and poor specificity. False positive elevations may occur in patients with renal impairments, older age, and low body mass index (BMI) [58]. Predictive biomarkers could greatly impact care, and guide preferred first line treatment selection by identifying patients who may have greater benefit from dual checkpoint inhibitor therapy to those who may have greater benefit from combined chemotherapy with immunotherapy. This necessitates the need for further exploration on reliable circulating predictive biomarkers.

### 4.1. Mesothelin and Soluble Mesothelin-Related Proteins

Serum mesothelin has attracted research interest as a response biomarker in individuals with PM. A multicenter prospective analysis found that elevated serum mesothelin is correlated to disease progression regardless of baseline serum mesothelin levels [59]. A 25% change in serum mesothelin from baseline serum mesothelin was the optimal threshold for disease progression, with a sensitivity of 48.7% and a specificity of 75.7%. While this is an improved sensitivity compared to mesothelin’s diagnostic biomarker sensitivity, it is too low to reliably detect progression of disease independently.

MESOMARK, which detects SMRP, is the only FDA-approved serum test and serves as a response biomarker for patients with PM. SMRP is associated with total tumor volume for PM, and levels have been found to decrease in individuals after surgical resection [32,60]. Recently, a phase I/II clinical trial studied SMRP levels in previously treated solid tumors including PM that were infused with a mesothelin-targeting T cell receptor fusion construct [61]. Decreased SMRP levels were seen in 21 out of 22 individuals, including 6 individuals who had responses to treatment. One clinical trial is currently evaluating SMRP levels as a companion biomarker in response to treatment for individuals undergoing autologous T cell therapy (NCT02414269) [62]. Currently, despite FDA approval, SMRP response assessment is not routinely used in clinical practice due to its influence from age, glomerular filtration rate, and BMI [58]. To better understand SMRP’s clinical utility, large randomized clinical trials will need to continue incorporating SMRP as a companion biomarker to investigate its correlation with response to specific systemic therapies.

SMRP has also been investigated as a potential prognostic biomarker. A meta-analysis of eight studies including 579 patients with PM found that elevated serum SMRP levels correlated with inferior OS [16]. Another study evaluated SMRP levels in a prospective immune checkpoint inhibitor clinical trial and found that lower levels (<1.35 nmol/L) were an independent prognostic factor that associated with improved OS [63]. However, further prospective studies are needed to validate SMRP’s prognostic utility across diverse treatment settings and histologic subtypes, establish the standardized thresholds for clinical interpretation, and clarify whether dynamic changes in SMRP levels can serve as a reliable prognostic indicator. Similarly to SMRP’s role as a response biomarker, large randomized clinical trials will need to incorporate SMRP as a companion biomarker to best understand its prognostic role.

### 4.2. KL-6

Krebs von del Lungan-6 (KL-6) is a human mucin-1 glycoprotein expressed in several malignancies in addition to PM, including lung, breast, ovarian, and pancreatic adenocarcinoma. KL-6 is released into circulation during alveolar epithelial injury from asbestos exposure, leading to elevated serum KL-6 levels. KL-6 serum concentrations have been found to be elevated in asbestosis-exposed workers compared to those exposed to silicosis or dust [64]. One study evaluated pleural fluid and serum KL-6 levels as potential diagnostic and prognostic biomarkers, both individually and in combination with SMRP [65]. Individuals with cytologically positive PM exhibited higher pleural effusion KL-6 levels compared with non-malignant controls, and these levels positively correlated with SMRP concentrations. Similarly, serum KL-6 levels demonstrated a significant correlation with serum SMRP levels. Among patients with PM, those with pleural effusion KL-6 levels greater or equal to 303 IU/mL had an improved median OS compared with those with lower KL-6 levels (23.0 vs. 9.2 months; HR 0.51 *p* = 0.004). While this study is promising, it only evaluated 87 patients, and expanded cohorts will be needed to confirm this biosignature.

### 4.3. Micro-RNA

In the MAPS trial, two miRNAs (miR-193b-3p and miR132-3p) were analyzed in 236 samples [17]. Low expression of miR193b-3p or miR-132-3p was associated with longer median OS in individuals with PM. Additionally, the MAPS trial also identified that low expression of mi-R-155-5p, miR-29c-5p, miR-132-3p, and miR-100-5p were predictive of improved survival in patients treated with bevacizumab and chemotherapy. This data is noteworthy and highlights promise for clinical application of specific miRNAs as potential prognostic biomarkers.

### 4.4. Eosinophils

Eosinophils have an integral yet complex role in the immune response of the tumor microenvironment (TME). The TME contributes to cancer progression and metastasis by providing a nest conductive for cellular growth [66]. Eosinophils largely interact with the TME through the release of cytokines as well as through direct contact with cancer cells, though this pathophysiology is poorly understood [67]. One retrospective analysis found that pre-treatment levels of peripheral eosinophils greater than 220 eosinophils per microliter (µL) of blood is associated with worse overall survival (14 vs. 29 months above and below 220 eosinophils/µL; HR 2.063, *p* = 0.0001) and shorter median PFS (8 vs. 17 months above and below 220 eosinophils/µL; HR 2.589, *p* < 0.0001) [68]. It is important to note that the normal range for eosinophils is between 0 and 450, and there is no clear mechanism to explain fluctuation within this range. Additionally, individuals who would require systemic treatments that could modulate the immune system, such as asthma, allergies, and autoimmune diseases, were excluded. These populations are important to incorporate, as many individuals with PM are prescribed medications that increase eosinophils such as beta-2 adrenergic receptor agonists, which are a mainstay in the management of many pulmonary disorders, including pulmonary fibrosis. Incorporation of these patients in prognostic biomarkers is necessary. Additionally, exclusion of this population could skew the correlation with eosinophils, especially given the threshold was within the expected range for a healthy control. Despite these limitations, this causal link does open potential ideas for prognostic implications as a biomarker for PM in addition to the incorporation of pre-treatment eosinophil suppression as a therapeutic strategy.

### 4.5. What Remains to Be Addressed

Currently, prognostic circulating biomarkers have several clinical applications that need to be assessed. Similarly to diagnostic circulating biomarkers, existing biomarkers lack the prognostic sensitivity and specificity needed to measure dynamic changes over time. Much of this is due to the limited data incorporating serial monitoring of prognostic biomarkers in large prospective cohorts. Long term follow-up is necessary for prognostic biomarkers since serial changes are tracked, which is unique when compared to diagnostic studies. A study including this is logistically cumbersome and requires a detailed prespecified framework to best assess the whether the biomarker prompts earlier imaging or influences treatment intensity. Additionally, while mi-RNA has begun to be studied in this space, there is limited data on ctDNA or epigenetic alterations. Studies incorporating these biomarkers, potentially as a prognostic signature may be clinically sensitive and specific in assessing progression and prognosis. 

## 5. Targeted Therapy

While much progress has been made in developing targeted therapeutics in NSCLC, advancements are limited in PM. As understanding of the tumorigenesis and genetic landscape of PM continues to expand, completed clinical trials (Table 2) and ongoing studies (Table 3) evaluating therapeutic strategies to target these molecular mechanisms are shown with a schematic diagram to represent highlighted biologic mechanisms (Figure 1).

### 5.1. Mesothelin Targeting Therapies

Amatuximab (MORab-009), a monoclonal antibody targeting mesothelin, was studied in a phase II trial in combination with pemetrexed and cisplatin for patients with unresectable PM. The regimen demonstrated a partial response rate of 40%, stable disease of 51%, and median PFS of 6.1 months [69]. Unfortunately, this study failed to meet its primary endpoint, as the PFS observed with combination chemotherapy showed no significant improvement compared with first-line treatment regimens. Treatment-emergent adverse events were notable for nausea (71%), fatigue (61%), anorexia (43%), constipation (30%), anemia (29%), neutropenia (29%), infusion-related reactions (12%), and hypersensitivity reactions (9%). Three patients discontinued treatment due to adverse events of hyperbilirubinemia, peritonitis, and cardiopulmonary arrest. The ARTEMIS trial, a phase II study evaluating the same combination in patients with unresectable PM, was subsequently terminated early due to business-related reasons [88].

Following the limited efficacy observed with monoclonal antibodies, attention shifted toward the development of antibody–drug conjugates targeting mesothelin. Anetumab ravtansine, a human anti-mesothelin antibody conjugated to a tubulin inhibitor named ravtansine, binds to mesothelin with high affinity and exerts a bystander effect on neighboring tumor cells [89]. Anetumab ravtansine was evaluated in a phase II multicenter study involving mesothelin positive (defined as 2+ or 3+ mesothelin membrane staining in at least 30% of viable tumor cells) patients with PM previously treated with platinum-based therapy [90]. The study failed to meet its primary endpoint, as the median PFS was similar between anetumab and vinorelbine (4.3 vs. 4.5 months, respectively). Adverse events reported in the anetumab ravtansine arm included nausea (42%), corneal disorder (37%), anorexia (33%), diarrhea (32%), dyspnea (14%), and peripheral neuropathy (12%). The most common grade 3 or greater treatment emergent events in the anetumab ravtansine arm included pneumonia (4%), neutropenia (1%), and dyspnea (6%). Phase II efforts to combine anetumab ravtansine with pembrolizumab in patients with PM found no difference in response rates compared to pembrolizumab monotherapy [91]. Bispecific antibodies were also briefly assessed in mesothelioma. A single-armed phase I study of ABBV-428, a CD40 and mesothelin-targeted bispecific antibody, was studied in patients with advanced solid tumors who progressed on or did not tolerate standard therapies [92]. Three out of nine patients had stable disease on treatment for greater than 6 months, but no responses were observed. Across all 59 patients treated with ABBV-428, seven patients had infusion-related reactions with grade 3 or greater treatment-related adverse events including pericardial effusion (2%), colitis (2%), infusion-related reaction (2%), and pleural effusion (2%).

Immunotoxins targeting mesothelin have attracted research interest in mesothelioma. SS1P is a recombinant, single-chain immunotoxin with the same single chain fragmented variable domain of amatuximab combined with a 38 kDa Pseudomonas exotoxin A, PE38 [93]. SS1P undergoes endocytosis after binding to mesothelin, causing PE38 to terminate protein synthesis and thereby facilitate apoptosis. A phase I dose-escalation study of SS1P enrolled 34 individuals with mesothelin-positive cancers, defined as ≥30% cells with mesothelin expression on IHC staining [94]. Among 20 patients with PM, 14 had stable disease, 2 had a minor response (defined as a decreased tumor area greater than 20% but less than 50% from baseline lasting four weeks), and 4 had progressive disease. Adverse events reported across all patients included hypoalbuminemia (61.8%), fatigue (55.9%), fever (24.3%), edema (24.5%), and abdominal pain (21.6%). SS1P was subsequently evaluated in combination with chemotherapy in a phase I trial consisting of 24 chemotherapy naïve individuals with unresectable epithelial or biphasic PM [95]. Individuals were treated with cisplatin, pemetrexed, and escalating doses of SS1P at four dose levels ranging from 25 to 55 mcg/kg. Of the 20 evaluable individuals, 12 had a partial response, 3 had stable disease, and 5 had progressive disease. The median PFS was 6.0 months, and the median OS was 13.6 months. Grade 3 toxicities across all individuals included hypoalbuminemia (21%), back pain (13%), and hypotension (8%). While the results were promising, the study had limited efficacy when compared to standard first-line therapies such as dual checkpoint inhibition at the time of analysis. Another immunotoxin, LMB-100, is a humanized anti-mesothelin with a 24 KDa de-immunized pseudomonas toxin, PE24. A phase I study of 10 previously treated individuals with PM demonstrated stable disease in 8 individuals at 28 days [96]. LMB-100 was also studied in combination with pembrolizumab for patients with PM; however, the study was terminated early due to the approval of ipilimumab and nivolumab (NCT03644550) [97]. There are no ongoing clinical trials evaluating immunotoxins registered with ClinicalTrials.gov, which is largely suspected to be due to the limited efficacy.

Chimeric antigen receptor (CAR) T cell therapies have emerged as potential treatment options for mesothelin-expressing tumors. Mesothelin-specific CAR-T therapy was studied in a phase I clinical trial consisting of two patients, one with PM and another with pancreatic cancer [98]. The anti-mesothelin CAR-T construct consisted of an anti-mesothelin single-chain variable fragment (scFv) with T cell costimulatory domain 4-1BB and T cell receptor zeta (TCR-ζ) signaling modules, which exhibit in vitro cell viability of 91.4%. The PM patient had a PFS of 6 months. Another phase I study of 5 patients with PM were treated with a lymphodepletion regimen of cyclophosphamide monotherapy followed by CAR-T infusion. The CAR-T product used an autologous T cell with an anti-mesothelin scFv with a cluster of differentiation 3 zeta (CD3 ζ) and 4-1BB costimulatory domains [99]. Individuals had minimal side effects, with low-grade fatigue and nausea observed in 47% of all patients in the study. The CAR-T products were able to expand in vitro; however, only four out of five individuals with PM had stable disease after 28 days. Three patients had progression at 3 months. There was a short persistence of CAR-T cells in vivo, which was hypothesized to be due to the cyclophosphamide lymphodepletion regimen. Combination of cyclophosphamide and fludarabine lymphodepletion has demonstrated superiority when compared to cyclophosphamide monotherapy in hematologic malignancies [100]. This is attributed to inducing a deeper and sustained lymphopenia to improve in vivo T cell expansion with combination fludarabine and cyclophosphamide. The study was potentially unsuccessful due to the combination of the lymphodepletion regimen and lack of CAR-T persistence of the product itself. In order to improve the poor T cell persistence in the blood, a study administered mesothelin-targeted CAR-T therapy intrapleurally in combination with intravenous pembrolizumab [70]. Regional delivery was found to be safe, and 8 out of 18 individuals had stable disease for greater than 6 months with a 1-year OS of 83%. While this slightly improved efficacy, regional delivery of cellular therapy can be challenging and limited to large academic institutions. Larger clinical trials are needed to demonstrate efficacy of intrapleural administration as well as understand methods to improve CAR-T cell therapy persistence. To combat T cell exhaustion, a phase I study evaluated BZDS1901, an armored mesothelin-targeted CAR-T that secretes a PD-1 nanoantibody when interferon-gamma is activated [71]. The study had 11 previously treated mesothelioma patients with positive PD-L1 expression and positive mesothelin expression (defined as greater than or equal to 50% of the mesothelin positive cells on IHC staining) in the tumor specimen. A lymphodepletion regimen of fludarabine and cyclophosphamide was administered prior to infusion of BZDS1901. Seven patients obtained an objective tumor response with one complete response and six partial responses. Four patients had stable disease. BZDS1901 was found to be safe with 54% of patients having lymphopenia that was reversible with supportive care. This study was promising given the safety and ORR of 63.6%. Larger trials investigating BZDS1901 in PM are warranted.

T cell receptor fusion constructs (TRuC) are endogenous T cell receptor subunits paired with a scFv to facilitate T cell binding and are being examined as options for individuals with mesothelin-expressing PM. This is different than CAR-T therapy, as the entire T cell receptor complex has an endogenous activation domain [101]. Mesothelin-targeted TRuC has demonstrated a reduction in tumor volume, enhanced tumor infiltration, and improved T cell persistence in vitro as well as in preclinical models [102]. Gavocabtagene autoleucel (gavo-cel) is an autologous TRuC engineered with a lentiviral vector to express an anti-mesothelin single domain antibody that is fused to the cluster of differentiation 3 epsilon (CD3ε) subunit. A phase I study evaluated gavo-cel in previously treated mesothelin-expressing (defined as 2+ or 3+ mesothelin expression by IHC staining in ≥50% of tumor cells) solid tumor patients, 23 of whom had PM [72]. Individuals received different dosing levels (5 × 10^7^−5 × 10^8^ cells/m^2^) of gavo-cel, either as a single agent or after fludarabine and cyclophosphamide lymphodepletion therapy. Individuals treated with lymphodepletion had an ORR of 21%, a median PFS of 5.6 months, and a median OS of 8.1 months [103]. Grade 3 or higher pneumonitis occurred in 16% of patients but none at the recommended phase 2 dose. Grade 3 or higher cytokine release syndrome occurred in 25% of patients but only 15% at the recommended phase 2 dose. There is an arm of the phase I/II trial evaluating the benefit of combination therapy with nivolumab or pembrolizumab in individuals with PM (NCT03907852) [77].

Mesothelin targeted bispecific antibodies, CAR-T products, and TRuCs have attracted research interest as potential therapeutic options for individuals with PM. CT-95, a bispecific antibody that engages both mesothelin and CD3, is currently being evaluated in patients with mesothelin-expressing PM (NCT06756035) [78]. A glypican-3 (GPC3) and mesothelin-targeted CAR-T therapy is under investigation in a phase I trial for individuals with PM (NCT06196294) [79]. TNhYP219, a mesothelin CAR-T product designed to target the membrane-proximal epitope of mesothelin, demonstrated efficacy in preclinical models, and is now being evaluated in a phase I trial (NCT06885697) [80,104]. Preclinical models have found a benefit of a mesothelin-targeting TRuC T cell that contains a chimeric switch receptor targeting PD-1 and CD28 [105]. The chimeric switch receptor is hypothesized to prevent T cell exhaustion by limiting chronic stimulation. An ongoing phase I/II trial is currently evaluating this TRuC chimeric switch receptor in individuals with 1+, 2+, or 3+ mesothelin expression in at least 50% of tumor cells by IHC (NCT05451849) [81]. In addition, anti-mesothelin CAR-NK cells, previously studied in cervical cancer, represent another potential therapeutic strategy in PM [106]. Preclinical models have shown benefit of a tri-specific T cell-activating construct composed of an anti-mesothelin antibody, a scFv against CD3, and an anti-albumin antibody coined HPN536 [107]. A phase I/IIa study of HPN536 in patients with mesothelin-expressing cancers, including PM, was recently completed, and the publication of these results is pending (NCT03872206) [82].

### 5.2. BRCA-1 Associated Protein 1 Targeting Therapies

BAP1 is a nuclear deubiquitinase protein that directly interacts with BRCA-1 associated ring domain protein 1 (BARD1) to inhibit the E3 ubiquitin ligase activity of BRCA-1/BARD1 [108]. BRCA-1/BARD1 complex regulates DNA damage response through homologous recombination and nucleotide excision repair [109]. HDAC1 assists in chromatin regulation and remodeling by removing acetyl groups from lysine residues to prevent transcription of genes [110]. BAP-1 depletion has been shown to increase HDAC1 levels in vitro in mesothelioma cells [111]. When BAP1 is lost, HDAC1 is hyperubiquinated, thereby losing its function, and homologous recombination is impaired. This leads to accumulation of DNA damage resulting in cellular necrosis and HMGB1 secretion, which promotes malignant transformation to PM [36]. While a majority of BAP1 mutations are somatic (60%), patients with PMs harboring germline BAP1 mutations have been shown to experience improved survival compared to those with somatic mutations [112]. Poly ADP-ribose (PARP) inhibitors have been studied in BAP1-deficient individuals with PM. PARP inhibitors are effective at killing tumor cells which lack the tumor suppressor activity of BRCA1/2 through synthetic lethality [113]. Olaparib was studied in a phase II trial in 23 patients with previously treated pleural and peritoneal mesothelioma [73]. The study showed limited efficacy, with 78% of individuals having stable disease after 6 weeks, an overall PFS of 3.6 months, and overall OS of 8.7 months. Individuals with somatic BAP1 mutations had a statistically significantly higher median PFS and median OS compared to those with germline mutations; however, the benefit was still comparable to traditional second line chemotherapy treatments. There were no new safety concerns with the use of olaparib in patients with PM. Rucaparib is another PARP inhibitor studied in a phase II trial for treatment refractory BAP1 deficient or BRCA-1 mutated mesothelioma [114]. In this study of 26 individuals with PM, 89% of patients were BAP1 negative, 50% were BRCA-1 mutated, and a total of 38% were both BAP1 negative and BRCA-1 mutated. The disease control rate (DCR) was 58% at 12 weeks and 23% at 24 weeks. Rucaparib demonstrated a median PFS of 17.9 weeks and a median OS of 41.4 weeks, which is comparable to second-line standard of care therapies. The most common grade 1–2 events were nausea (69%), fatigue (54%), and decreased appetite (38%). The most common grade 3–4 adverse events were upper respiratory tract infection (12%) and anemia (12%). Niraparib was studied in a phase II trial of 17 previously treated individuals with solid tumors who were likely to be BAP1 negative, which was defined as those with a prespecified DNA damage response repair mutation [115]. Three individuals with PM were included, and two of these patients harbored a BAP1 mutation upon confirmatory testing. Of the two patients, one demonstrated a partial response but had to withdraw from the trial due to grade 2 confusion and grade 3 tremor, while the other patient had progressive disease. Overall, PARP inhibitors largely showed limited efficacy in BAP1-negative patients. This is likely because BAP1 has a primary role of inhibiting E3 ubiquitin ligase but is not a core DNA repair protein and thus does not impair homologous recombination repair similarly to individuals with BRCA mutations.

Enhancer of zeste-homolog 2 (EZH2) inhibitors have likewise been the subject of investigation in BAP1-deficient individuals with PM. EZH2 is a catalytic subunit that leads to chromatin remodeling and repression of transcription. Tazemetostat was studied in a phase II trial of 74 previously treated individuals with PM, 73 of whom had a BAP1 depletion [116]. Disease control was achieved in 54% of patients, and only 2 patients had a partial response. The median PFS was 18 weeks, and the median OS was 36 weeks. Grade 3–4 adverse events occurred in 34% of patients, most commonly hyperglycemia (7%), hyponatremia (7%), and anemia (5%). The modest efficacy of tazemetostat, coupled with its toxicity profile, likely explains the lack of further studies for EZH2 inhibitors in PM. Vorinostat, a HDAC inhibitor, was studied in a phase III trial of 329 previously treated individuals with PM, comparing vorinostat to placebo [117]. Individuals had a median OS of only 30.7 weeks, which was similar to individuals treated with a placebo.

### 5.3. NF2 Targeting Therapies

*NF2* encodes for merlin, which is a tumor suppressor protein inactivated in malignant mesothelioma and is found in about 40% of individuals with PM [118]. Merlin activates the Hippo signaling pathway to induce accumulation of underphosphorylated yes-associated protein (YAP) and tafazzin (TAZ) in the nucleus [119]. YAP and TAZ bind to the transcriptional enhanced associated domain (TEAD) and upregulate transcription of oncogenic genes to enhance proliferation, cellular migration, and loss of contact inhibition. NF2 loss is typically associated with a more aggressive phenotype but does not commonly stimulate the direct transformation to PM [120]. A phase I study of 29 individuals with PM evaluated the efficacy of VT3989, an oral inhibitor of TEAD palmitoylation and the first in its class to inhibit YAP/TEAD activation [74]. Eight patients achieved a partial response, and three of them had partial responses lasting longer than 18 months. Eight grade 3 adverse events (fatigue, ALT, AST, dehydration, dyspnea, hypotension, cardiomyopathy, and peripheral edema) were reported. Phase II data of VT3989 was published and recently presented at ESMO 2025 [121]. The study included 172 patients, 135 of whom had heavily pretreated mesothelioma regardless of *NF2* mutational status. The ORR was 26% in the 47 mesothelioma patients with available outcomes data. Of the 22 mesothelioma patients treated with optimized dose levels of VT3989, the ORR was 32% with 7 having partial responses and 12 having stable disease. DCR was 86% and mPFS was 10 months. The subgroup analysis for patients with PM has not yet been reported. This is promising data which led to VT3989 being awarded orphan drug designation and fast track designation by the FDA. The safety profile for mesothelioma patients was favorable with predominantly low-grade toxicities, including increased urine albumin/creatinine ratio (31.4%), proteinuria (27.9%), peripheral edema (23.3%), and fatigue (19.8%). VT3989 is now being studied in combination with immunotherapy (nivolumab plus ipilimumab) in individuals with PM with or without *NF2* mutations (NCT04665206) [83]. IK-930 is another oral inhibitor of TEAD palmitoylation that was studied in a phase 1 clinical trial but was terminated due to sponsor strategic regions [122]. There are several ongoing clinical trials evaluating YAP/TEAD inhibitors as a therapeutic option in individuals with PM. IAG933, a small molecular that disrupts the interaction of YAP and TEAD proteins, is currently being studied in a phase I clinical trial in patients with advanced mesothelioma (NCT04857372) [84]. SW-682 is a novel pan-TEAD inhibitor that blocks TEAD-dependent transcription by binding to the palmitoylation pocket, and it showed benefit in preclinical models [123]. SW-682 is currently being studied in a phase I clinical trial for individuals with mesothelioma with or without *NF2* mutations (NCT06251310) [85].

### 5.4. Other Molecular Targeting Therapies

*Cyclin Dependent Kinase Inhibitor 2A* (*CDKN2A*) is a gene that encodes p16 and inhibits cyclin-dependent kinase (CDK) 4 and CDK6 expression, limiting retinoblastoma protein phosphorylation during the G1/S cell cycle checkpoint [124,125]. *CDKN2A* homozygous deletions allow DNA-damaged cells to proliferate, thus leading to mesothelial transformation. Inhibiting CDK4 and CDK6 has shown efficacy in preclinical PM models [126,127]. Abemaciclib, a CDK4/CDK6 inhibitor, was studied in 27 previously treated individuals with p16-deficient PM in a single-arm phase II trial and showed a modest benefit with a 54% DCR at 12 weeks [128]. Two individuals had a partial response, and 11 individuals had stable disease. A greater reduction in tumor volume was seen in patients with *CDKN2A* and S-methyl-5’-thioadenosine Phosphorylase (MTAP) codeletions, which may suggest a greater clinical benefit in this subset. The safety profile was notable for 3 patients with grade 3 or greater treatment-related adverse events (diarrhea, thrombocytopenia, pulmonary embolism, elevated ALT, and vomiting). Further investigation of combination chemotherapy and abemaciclib has demonstrated durable antiproliferative effects in vitro [129]. Inhibition of CDK4/6 and PI3K inhibitors have also demonstrated efficacy in preclinical models [130]. Future clinical trials using CDK4/CDK6 in combination with other systemic therapy could potentially be explored despite modest results with abemaciclib monotherapy.

MTAP is the rate-limiting enzyme for the salvage synthesis of methionine and adenine [131]. Deficiency or inhibition of MTAP leads to a buildup of methylthioadenosine (MTA) which binds to protein arginine methyltransferase 5 (PRMT5) and inhibits its ability to methylate genes [132]. Individuals with MTAP deletions have reliance on PRMT5 for gene methylation, and inhibition can result in the loss of methylation, resulting in cell death from synthetic lethality [133]. Inhibition of PRMT5 may be a therapeutic target for MTAP-deleted tumors. One phase I/II study is assessing TNG908, an oral small molecule that inhibits PRMT5, in MTAP-deleted tumors and includes individuals with PM (NCT05275478) [86,134]. MRTX1719, an oral small molecule that selectively inhibits the PRMT5/MTA complex, is being studied in a phase I clinical trial in patients with homozygous MTAP-deleted solid tumors (NCT05245500) [87].

Anexelekto (AXL) is a transmembrane receptor that binds to Gas-6 to activate PIK3/AKT, extracellular signal-regulated kinase (ERK), mitogen-activated protein kinase (MAPK), and signal transducer and activator of transcription (STAT) pathways [135]. AXL overexpression can be found in up to 75% of PMs and functions by triggering cell proliferation, survival, and invasion of cancer cells. MiST3 is a phase II study of bemcentinib, a selective AXL tyrosine kinase inhibitor, in combination with pembrolizumab in 26 previously treated individuals with PM [136]. Combination therapy demonstrated a DCR of 46.2% at 12 weeks, DCR of 38.5% at 24 weeks, and ORR of 15.4%. Grade 3 or greater treatment related adverse events were found in 38% of patients, and the most common adverse events were fatigue (46%) and nausea (42%). While the ORR is similar to historical single-agent chemotherapy response rates, bemcentinib could be further evaluated in combination with chemotherapy. To further explore potential combination therapies for AXL, one study demonstrated favorable efficacy for dual inhibition of AXL and ataxia telangiectasia and rad3-related (ATR) protein in PM cells in vitro and in xenograft models [137]. One xenograft model showed a statistically significant decrease in tumor growth with dual inhibition of AXL and ATR when compared to xenograft models treated with either drug alone. Combining AXL and ATR inhibition could be a promising therapeutic approach that needs to be evaluated in future clinical trials.

Arginine Succinate Synthetase 1 (ASS1) is the rate-limiting enzyme in the production of arginine, an amino acid important in cell proliferation. Depletion or loss of expression of ASS1 is seen in 63% of PMs and facilitates increased cell proliferation and enhanced cell migration, and is predominantly found in sarcomatoid and biphasic histologic subtypes [138]. One phase II clinical trial enrolled 68 previously treated individuals with PM and ASS1 deficiency (defined as <50% of cells positive for ASS1 on IHC staining) to receive intramuscular injections of pegylated arginine deiminase (ADI-PEG20) plus best supportive care or best supportive care alone [139]. The ADI-PEG20 group showed a statistically significant improvement in median PFS (3.2 vs. 2.0 months; HR 0.56, *p* = 0.03). Treatment-related adverse events in the ADI-PEG20 group included abnormal hematologic test result (21%), allergic reaction (11%), injection site reaction (20%), chest pain/difficulty breathing (16%), gastrointestinal events (14%), fatigue (16%), and rash (16%). Given this PFS benefit, ADI-PEG20 was further studied in combination with platinum-based chemotherapy vs. chemotherapy alone in the ATOMIC-meso trial [75]. This was a phase II/III double-blind clinical trial where 249 treatment naïve non-epithelioid PM patients were randomly assigned to receive ADI-PEG20 or placebo until progression in combination with pemetrexed and platinum-based chemotherapy for up to six cycles. The ADI-PEG20-chemotherapy group showed a statistically significant improvement in median PFS (6.2 vs. 5.6 months; HR 0.65) and median OS (9.3 vs. 7.7 months; HR 0.71) compared to chemotherapy alone. Grade 3 to 4 adverse events occurred in 28.8% of individuals treated with ADI-PEG20 and 16.9% of individuals treated with a placebo. Grade 3 or greater treatment-related events in the combination arm included anemia (4.8%), neutropenia (4.8%), thrombocytopenia (3.2%), nausea (1.6%), rash (1.6%), fatigue (1.6%), and hyponatremia (1.6%). One particular limitation of this study was that twice as many patients in the ADI-PEG20-chemotherapy group received ipilimumab and nivolumab immunotherapy upon progression, which potentially affected long-term survival. There appears to be less of a survival benefit with ADI-PEG20 plus chemotherapy compared to ipilimumab and nivolumab in the first-line setting in CheckMate-743 based on cross trial comparisons. Further clinical studies evaluating ADI-PEG20 and chemotherapy in combination with checkpoint inhibitors may be informative to determine the role of ADI-PEG20 in treating patients with sarcomatoid PM.

The Wilm’s tumor gene 1 (*WT1*) encodes for the WT1 protein, a nuclear and cytoplasmic zinc-finger protein which is crucial in the embryonic development and differentiation of the kidneys, spleen, and mesothelium but is typically silenced in adult tissues [140]. WT1 overexpression promotes the epithelial to mesenchymal transition and resistance to apoptosis in PM cell lines [141,142]. Researchers have designed synthetic analog peptides to inhibit WT1. Galinpepimut-S (GPS), a WT-1 analog peptide vaccine, functions by generating cross-reactivity to native peptides to elicit WT1-specific T cell recognition [143]. GPS was studied in 41 patients with resected PM as adjuvant therapy and demonstrated a favorable safety profile with any grade treatment-related events including injection site reactions (85%), fatigue (50%), and nausea (10%) [76]. The study had a median PFS of 10.1 months and a median OS of 22.8 months. While this data is promising, studies evaluating adjuvant therapy for mesothelioma are limited, and a randomized trial comparing GPS and chemotherapy would help better understand its efficacy. Another study evaluated GPS in combination with nivolumab in individuals with WT1-expressing previously treated PM [144]. Ten patients were enrolled and treated with two doses of GPS followed by six doses of GPS with nivolumab every 2 weeks. Three patients had stable disease for up to 4 months, and a 17% average decrease in tumor volume was observed. No partial responses were observed. The median PFS was 3.9 months, and the median OS was 7.4 months. Combination therapy demonstrated no radiographic activity in individuals with resected PM. The most common treatment-related adverse events reported were fatigue (20%), infusion-related reaction (20%), and skin induration (20%). The authors hypothesize the limitations in efficacy of GPS with nivolumab to be due to insufficient vaccine-specific humoral responses.

MicroRNA play a role in the regulation of gene expression and are attracting attention for their role in tumor suppression. MiR-3140-3p is a miRNA that suppresses tumor cell proliferation in PM cancer cell lines, leading to cellular senescence and death [145]. MIRX002 is the nucleic acid miR-3140-3p and formulated with A6K, a delivery peptide for administration. It is currently undergoing phase 1 investigation at Hiroshima University in Japan [146].

### 5.5. What Remains to Be Addressed

Targeted therapies have a large potential to aid unmet gaps in the treatment of PM; however, advancement in therapeutics has been limited. Mesothelin targeted therapies have been the most heavily studied yet unsuccessful. Questions remain if the limited efficacy is due to the lack of potent therapeutic options seen in studies with monoclonal antibodies, ADCs, and cellular therapy, or whether incorporation of predictive biomarkers will help better define which population of PM patients would most benefit. BAP1 deficiency has also showed limited efficacy when being treated with PARP and EZH2 inhibitors. While studies show that synthetic lethality through DNA damage repair targeted approaches are efficacious in other cancers with BRCA1 and BRCA2 mutations, these agents have not demonstrated efficacy for BAP1 deficient PM. This is due to the partial defect in homologous recombination rather than full defect seen with BRCA1 and BRCA 2 mutations. Incorporation of novel distinct therapeutic strategies to target BAP1 deficiency may improve clinical response. VT3989 has shown promise, and further studies of YAP/TAZ and TEAD inhibitors in specific patient populations will be interesting in the upcoming years. Lastly, there are several promising therapeutic targets on the horizon. Synthetic lethality through MTAP loss has yet to be addressed. Combining targeted therapies with immunotherapy, anti-angiogenic therapy, or with other targeted therapies has yet to be explored and could provide clinical benefit in biologic mechanisms that have largely been untargetable. 

## 6. Translational and Future Directions

Mesothelioma remains a challenging disease to diagnose due to its non-specific symptoms at presentation, radiographic complexity, and patchy tumor distribution. It is also difficult to treat due to its presentation at an advanced stage, aggressive nature, and limited treatment options. Circulating biomarkers are under extensive investigation for their potential role in diagnosing PM, assessing prognosis, monitoring treatment response, and discovering novel therapeutic targets. Unfortunately, no circulating markers have shown enough evidence for FDA approval as a diagnostic biomarker for PM. SMRP is the only FDA-approved response biomarker in mesothelioma, but it is not clinically utilized due to its low sensitivity for mesothelioma and false-positive rate from impaired renal function, older age, and low BMI. It has demonstrated promising improvements in diagnostic specificity when combined with other mutations such as the *mesothelin rs1057147* polymorphism as well as miRNA such as miR-126; however, it still lacks the sensitivity needed to be a valuable diagnostic biomarker. A study of 42 cases of positive SHOX2 and PTGER4 methylation combined with elevated serum CYFRA 21-1 demonstrated an impressive sensitivity (91.3%) and specificity (97.6%) in differentiating mesothelioma from reactive mesothelial hyperplasia; however, this diagnostic biomarker signature needs to be validated in a large prospective trial [15]. Incorporation of genetic methylation with circulating biomarkers can potentially be promising in assisting with the diagnosis of cytologically negative PM.

Mesothelioma has a recurrence rate of approximately 60% after resection, and it is currently unclear who is at an elevated risk for recurrence [147]. Identifying circulating biomarkers for postoperative minimal residual disease (MRD) assessment could allow for early detection of relapse and could inform treatment intensification in the adjuvant setting. One study evaluated patient-specific chromosomal rearrangements found in cell-free DNA (cfDNA) as an assessment for MRD [148]. Seven patients (three with peritoneal and four with pleural mesothelioma) were each selected to have three to five chromosomal junctions followed after completion of surgical resection. cfDNA was able to be assessed in four out of five patients with PM and serially monitored in one patient. The 3 PM patients without evidence of disease after surgery did not have detectable cfDNA after resection. In the one case of PM with detectable disease, the patient had a mixed response to treatment, with improvement in pleural disease and development of osseous metastases which correlated with persistent cfDNA detection at follow-up. While this is promising, the study had two additional patients who were unable to have their cfDNA assessed, including one with extensive biphasic PM which is thought to be due to the lack of tumor DNA shedding into circulation. Additionally, the sample size was small with only one PM patient serially monitored, which was attributed to the study being performed during the coronavirus disease 2019 pandemic. While this study provides evidence that a personalized serial monitoring of cfDNA is feasible, larger studies are necessary to demonstrate an association with MRD.

Immunotherapy has become a mainstay in the treatment of PM. At ESMO 2025, data from the DREAM3R trial was presented and did not show a statistically significant OS benefit with the addition of durvalumab to chemotherapy in patients with unresectable PM [149]. It is unclear if the lack of benefit with durvalumab was due to the heterogenous patient population in this study. Analysis of correlative biomarkers are pending. Incorporating circulating biomarkers into trials may help identify and select patients who are more likely to have favorable responses to immunotherapy. At this time, predictive biomarkers in mesothelioma are lacking, and incorporation into future clinical trial design is essential.

Since the FDA approval of pemetrexed in mesothelioma in 2004, no new therapies were approved until the combination of nivolumab and ipilimumab received approval in October 2020 based on results of CheckMate-743, followed by approval of pembrolizumab in combination with platinum-based chemotherapy and pemetrexed in September 2024 based on results of KEYNOTE-483 [150,151,152]. There is currently a clinical dilemma of distinguishing which first-line treatment strategy should be used for patients with PM. Traditional PD-L1 biomarkers and SMRP did not demonstrate correlation with response to immunotherapy in the KEYNOTE-483 subgroup analysis [7]. Currently, determining treatment for first-line therapy is largely based on histologic subtype, performance status, and patient comorbidities. Integration of circulating biomarkers may facilitate a tailored approach when selecting first-line therapy for patients with PM.

Despite these recent advances in immunotherapy, progress in developing targeted treatments for mesothelioma has remained slow. Environmental exposure from asbestos induces chromosomal damage and genomic region losses often leading to deletion of tumor suppressor genes such as *BAP1*, *NF2*, and *CDKN2A* [153]. This mutational landscape of mesothelioma poses a major challenge to therapeutic development, as it lacks actionable oncogenic drivers. Unlike oncogenic drivers that can be inhibited, tumor suppressor genes require their function to be restored or replaced, which is more difficult to achieve in drug development. The use of gene editing therapies such as CRISPR-based genomics has yet to be investigated and could provide potential benefit. Future directions can include using genetic therapy to introduce a copy of the lost tumor suppressor gene or to expose vulnerabilities through synthetic lethality such as in the MTAP/PMRT5 molecular pathway. Additionally, loss of tumor suppressor genes also increases tumor heterogeneity, as seen in mesothelioma, thereby affecting drug efficacy due to various subpopulations harboring different mutations [154]. Clinically relevant preclinical models to capture this heterogeneity are difficult to create but could provide great benefit in assessing novel targeted therapy options. 

There are several drugs that are currently in development with promising results. For instance, VT3989, a YAP/TEAD inhibitor, was recently granted FDA fast track designation for unresectable mesothelioma [155]. Clinical investigation into various treatment modalities such as vaccines with dendritic cells and oncolytic viruses in combination with chemotherapy or targeted therapy may provide clinical benefit [156]. Additionally, bispecific antibodies, trispecific antibodies, CAR-T products, and TRuC products are novel drugs targeting mesothelin. Chimeric switch receptor TRuCs present a potential solution to decrease T cell exhaustion in solid malignancies. Drugs targeting cell surface proteins such as AXL warrant further clinical investigation in combination with other therapies as this may represent a more effective approach in the treatment of PM.

As knowledge of the mutational landscape of PM expanded, this has led to the study of novel targeted therapies in PM and their combination with other modalities of systemic therapy. While there are no targeted therapies currently approved for the treatment of PM, ongoing research in PM will hopefully lead to the development of effective targeted therapeutics or approaches that will improve the lives of patients with mesothelioma.

## Figures and Tables

**Table 1:** Highlighted completed trials in circulating biomarkers.

Biomarker	Study	Matrix	N	Diagnostic/Prognostic Biomarker	Results
Mesothelin	Johnen [10]	Serum	23	Diagnostic	Sensitivity 23%, specificity 99% at cutoff 2.00 nmol/L
SMRP	Hollevoet [11]	Serum	4491	Diagnostic	Average study sensitivity 47%, specificity 96% when all studies were recalculated at standardized cutoff 2.00 nmol/L
SMRP + *Mesothelin rs1057147* positive	Goricar [12]	Serum	154	Diagnostic	Sensitivity 59.3%, specificity 93% at cutoff 1.5 nmol/L
Fibulin-3 + Mesothelin + HMGB1	Ferrari [13]	Serum	26	Diagnostic	Sensitivity 96%, specificity 93% without defined cutoffs for each biomarker reported
DNA repeats 115 bp, 247 bp, 247/115 bp ratio	Sriram [14]	Pleural/Serum Matched	52	Diagnostic	Sensitivity 81%, specificity 87% in combined index
CYFRA-21-1+ *SHOX2* methylation + *PTGER4* methylation	Zhang [15]	Serum	42	Diagnostic	Sensitivity 91.3%, specificity 97.6% without defined cutoff
SMRP	Tian [16]	Serum	579	Prognostic	Shorter overall survival (HR 1.958, 95% CI 1.531–2.504, *p* = 0.000)
MiR-193b-3p + MiR-132-3p	Levallet [17]	Serum	236	Prognostic	Shorter overall survival (HR 0.87 95% CI 0.81–0.93, *p* < 0.001) in those with low expression levels without defined cutoffs

Abbreviations: CYFRA-21-1, cytokeratin 19 fragment antigen 21-1; HMGB1, high-mobility group box 1; MiR-132-3p, microRNA-132-3p; MiR-193b-3p, microRNA-193b-3p; SMRP, soluble mesothelin-related protein.

**Table 2:** Highlighted completed molecular target trials in pleural mesothelioma.

Target	Study	Phase	N	Drug (s)	Efficacy
Mesothelin	Hassan [69]	II	89	Amatuximab + Cisplatin/Pemetrexed	40% PR; 51% SD; mPFS 6.1 months; mOS 14.8 months
Mesothelin	Adusmilli [70]	I	18	Mesothelin CAR T cell therapy administered intrapleurally + intravenous Pembrolizumab	44.4% SD at 6 months; OS 83% at 12 months
Mesothelin	Liu [71]	I	11	BZDS1901 (armored CAR T cell therapy with PD-1 nanoantibody secretion)	63.64% ORR; 54.5% PR; 9.1% CR
Mesothelin	Smalley [72]	I	23	Gavocabtagene autoleucel	21% ORR; mPFS 5.6 months; mOS 8.1 months
BAP-1	Ghafoor [73]	II	23	Olaparib	78% SD at 6 weeks; overall PFS 3.6 months; overall OS 8.7 months
NF2	Yap [74]	II	22	VT3989	32% ORR, 54.5% SD, mPFS 10 months when accounting for urine albumin:creatinine ratio at clinically optimized doses
ASS1	Szlosarek [75]	II/III	249	ADI-PEG20 + Platinum-based/Pemetrexed	mPFS 6.2 months; mOS 9.3 months
WT1	Zauderer [76]	II	41	Galinpepimut-S peptide vaccine after resection	mPFS 10.1 months; mOS 22.8 months

Abbreviations: ADI-PEG20, pegylated arginine deiminase; ASS1, arginine succinate synthetase 1; BAP1, BRCA-1 associated protein 1; CAR, chimeric antigen receptor; SD, stable disease; mOS, median overall survival; mPFS, median progression-free survival; ORR, overall response rate; PD-1, programmed cell death protein 1; PR, partial response; NF2, neurofibromatosis type 2; WT, Wilm’s tumor 1.

**Table 3:** Ongoing molecular target trials in pleural mesothelioma.

Target	Study	Phase	Anticipated Accrual	Drug (s)	Primary Endpoint (s)
Mesothelin	NCT03907852 [77]	I/II	57	Gavocabtagene autoleucel + Nivolumab or Ipilimumab	Dose-limiting toxicity; ORR + SD at 8 weeks; ORR at 3 months
Mesothelin	NCT06756035 [78]	I	70	CT-95 (mesothelin/CD3 bispecific T cell)	Dose-limiting toxicity
Mesothelin	NCT06196294 [79]	I	30	Mesothelin and Glypican-3 targeted CAR T cell	Dose-limiting toxicity, response rate at 6 months, T cell persistence
Mesothelin	NCT06885697 [80]	I	100	TNhYP218 (CAR T cell)	Dose limiting toxicity, response rate at 4, 8, 12 weeks, and until progression
Mesothelin	NCT05451849 [81]	I/II	6	TC-510 (TRuC PD-1/CD28 chimeric switch receptor)	Dose limiting toxicity, ORR at 8 weeks and until progression
Mesothelin	NCT03872206 [82]	I/II	95	HPN536 (Tri-specific CD3/anti-albumin/anti-mesothelin antibody)	Maximal tolerated dose, ORR at 1 year
NF2	NCT04665206 [83]	I/II	336	VT3989 (oral inhibitor of TEAD) + nivolumab + ipilimumab	Incidence of adverse events, tumor response
NF2	NCT04857372 [84]	I	137	IAG933 (small molecule inhibitor of YAP/TEAD)	Safety and maximally tolerated dose
NF2	NCT06251310 [85]	I	186	SW-682 (small molecule pan-TEAD inhibitor)	Safety and maximally tolerated dose, ORR
MTAP	NCT05275478 [86]	I/II	192	TNG908-C101 (oral small molecule inhibitor of PRMT5)	Safety and maximally tolerated dose
MTAP	NCT05245500 [87]	I	320	MRTX1719 (oral small molecule binding to PRMT5/MTA complex)	Dose limiting toxicity, ORR, duration of response, PFS, and OS

Abbreviations: CD3, cluster of differentiation 3; CD28, cluster of differentiation 28; MTA, methylthioadenosine; MTAP, methylthioadenosine phosphorylase; NF2, neurofibromatosis type 2; ORR, overall response rate; OS, overall survival; PD-1, programmed cell death protein 1; PFS, progression free survival; PRMT5, protein arginine methyltransferase 5; SD, stable disease; TEAD, TEA domain transcription factor; TRuC, T cell receptor fusion construct; YAP, yes-associated protein.

## Abbreviations

The following abbreviations in alphabetical order:

ADI-PEG20	Pegylated arginine deiminase
ASS1	Arginine succinate synthetase 1
ATR	Ataxia telangiectasia and rad3-related
AXL	Anexelekto
BAP1	BRCA-1 associated protein
BARD1	BRCA-1 associated ring domain protein
BMI	Body mass index
CAR	Chimeric antigen receptor
CD3 ζ	Cluster of differentiation 3 zeta
CD3ε	Cluster of differentiation 3 epsilon
CDK	Cyclin-dependent kinase
CEA	Carcinoembryonic antigen
cfDNA	Cell-free DNA
CtDNA	Circulating tumor DNA
CTLA-4	Anti-cytotoxic T-lymphocyte-associated antigen 4
CYFRA-21-1	Cytokeratin 19 fragment antigen 21-1
DCR	Disease control rate
DFS	Disease-free survival
ERK	Extracellular signal-regulated kinase
EZH2	Enhancer of zeste-homolog
FDA	Food and drug administration
Gavo-cel	Gavocabtagene autoleucel
GPC3	Glypican-3
GPS	Galinpepimut-S
HDAC1	Histone deacetylase 1
HMGB1	High-mobility group box 1
IHC	Immunohistochemical
KL-6	Krebs von del Lungan-6
MAPK	Mitogen-activated protein kinase
MAPS	Mesothelioma avastin pemetrexed study
MiRNA	Micro-RNA
MPF	Megakaryocyte potentiating factor
MRD	Minimal residual disease
MTA	Methylthioadenosine
MTAP	S-methyl-5′-thioadenosine phosphorylase
NSCLC	Non-small cell lung cancer
ORR	Overall response rate
OS	Overall survival
PARP	Poly ADP-ribose
PD-1	Programmed cell death 1
PFS	Progression-free survival
PM	Pleural mesothelioma
PRMT5	Protein arginine methyltransferase 5
RECIST	Response evaluation criteria in solid tumors
ScFv	Single-chain variable fragment
SMRP	Soluble mesothelin-related protein
STAT	Signal transducer and activator of transcription
TAZ	Tafazzin
TCR- ζ	T cell receptor zeta
TEAD	Transcriptional enhanced associated domain
TME	Tumor microenvironment
TRuC	T cell receptor fusion construct
WT1	Wilm’s tumor gene 1
YAP	Yes-associated protein
